# Predation Determines Different Selective Pressure on Pea Aphid Host Races in a Complex Agricultural Mosaic

**DOI:** 10.1371/journal.pone.0055900

**Published:** 2013-02-07

**Authors:** Adalbert Balog, Oswald J. Schmitz

**Affiliations:** 1 School of Forestry and Environmental Studies, Yale University, New Haven, Connecticut, United States of America; 2 Department of Horticulture, Faculty of Technical Science, Sapientia University, Tirgu Mures, Romania; Emory University, United States of America

## Abstract

Field assessments were conducted to examine the interplay between host plant and predation in complex agricultural mosaic on pea aphid clover and alfalfa races. In one experiment, we examined the relative fitness on clover race (CR) and alfalfa race (AR) pea aphids on broad bean, red clover and alfalfa alone. But because clover is typically grown in a more complex agricultural mosaic with alfalfa and broad bean, a second experiment was conducted to assess the fitness consequences under predation in a more complex agricultural field setting that also included potential apparent competition with AR pea aphids. In a third experiment we tested for the effect of differential host race density on the fitness of the other host race mediated by a predator effect. CR pea aphids always had fitness losses when on broad bean (had lower fitness on broad bean relative to red clover) and fitness benefits when on red clover (higher fitness on red clover relative to broad bean), whether or not in apparent competition with alfalfa race aphids on bean and alfalfa. AR suffered fitness loss on both alfalfa and bean in apparent competition with CR on clover. Therefore we can conclude that the predation rate between host races was highly asymmetrical. The complexity of the agricultural mosaic thus can influence prey selection by predators on different host plants. These may have evolutionary consequences through context dependent fitness benefits on particular host plants.

## Introduction

Speciation in phytophagous insects has earned significant attention in recent years [1]–[3]. This is because the high diversification of this group of animals may partly result from adaptive processes that reflect their specialization to different host plants (i.e., higher affinity to feed and reproduce on a particular plant) and associated assortative behavior among individuals within a group on one host plant-type potentially leads to reduced gene flow between groups feeding on different host plants [3]–[5]. In such cases, insect species form “host races” that are often a first step toward eventual sympatric speciation [3], [6]. These host races form, potentially leading to divergent selection via reproductive isolation [3], [7]. However, the mechanistic basis for the divergent selective pressure remains uncertain in many cases [8]. It has been suggested that behavior has a special importance in that it often initiates the use of a new environment—e.g., a new host plant for phytophagous insects—whereupon selection acts differently on individuals within the old and new environment [9]. A potentially important determinant of both behavior and diversifying selection in insects is the presence of predators [10]. Continuous predator threat can provoke population-wide phenotypic (behavioral, morphological and physiological) responses of prey species that leads to differential performance and fitness in different environmental contexts [11]–[15]. When predation pressure changes temporally, spatially (e.g., in a new environment or host plant), or qualitatively, those prey individuals that are able to respond flexibly to risk are predicted to have higher survival chances and subsequent fitness advantages [16], [17].

Here, we explore the potential for predation to be a determinant factor for differences in the performance of host adapted pea aphid (*Acyrthosiphon pisum*) populations. This species is considered a useful model to identify how divergence in host use may have arisen [6], [18], [19]. Multiple host races (13 are currently known) have been genetically distinguished globally. These populations were then introduced to North America [3], [20], [21]. The clover race (CR) and alfalfa race (AR) are among the least genetically differentiated pairs of host races in the pea aphid complex [22], [23]. Pea aphid feed by penetrating plant organs, such as the leaf or stem, with their stylets in search of vascular tissues [8]. The chemical and/or physical attributes of different plant tissues may make aphids reluctant to accept a new host plant because host selection involves a trade-off between preference for phloem sap and avoiding the need to deal with novel plant structures or chemicals encountered during the search for the phloem [24]. This may be overcome when pea aphids face predation. Many predators, including lady beetles, damsel bugs, hover flies, and parasitoid wasps, are known to attack pea aphids [25]–[27], but their susceptibility to natural enemies varies with host plant [28]. However, the relative performances of pea aphids on different hosts in the face of predatory pressure remains incompletely understood. We hypothesized that: 1. There is different predation pressure on CR and AR pea aphids on different agricultural mosaics (host plant combinations) and this can be reflected by increasing or decreasing fitness of pea aphids on these host plants. 2. Possible predator-induced apparent mutualism or competition between pea aphid host races may enhance fitness of one of the host races when in close proximity to the other host race. There are currently no studies showing that differential predation rate exists from a single natural enemy in the pea aphid complex.

**Figure 1:**
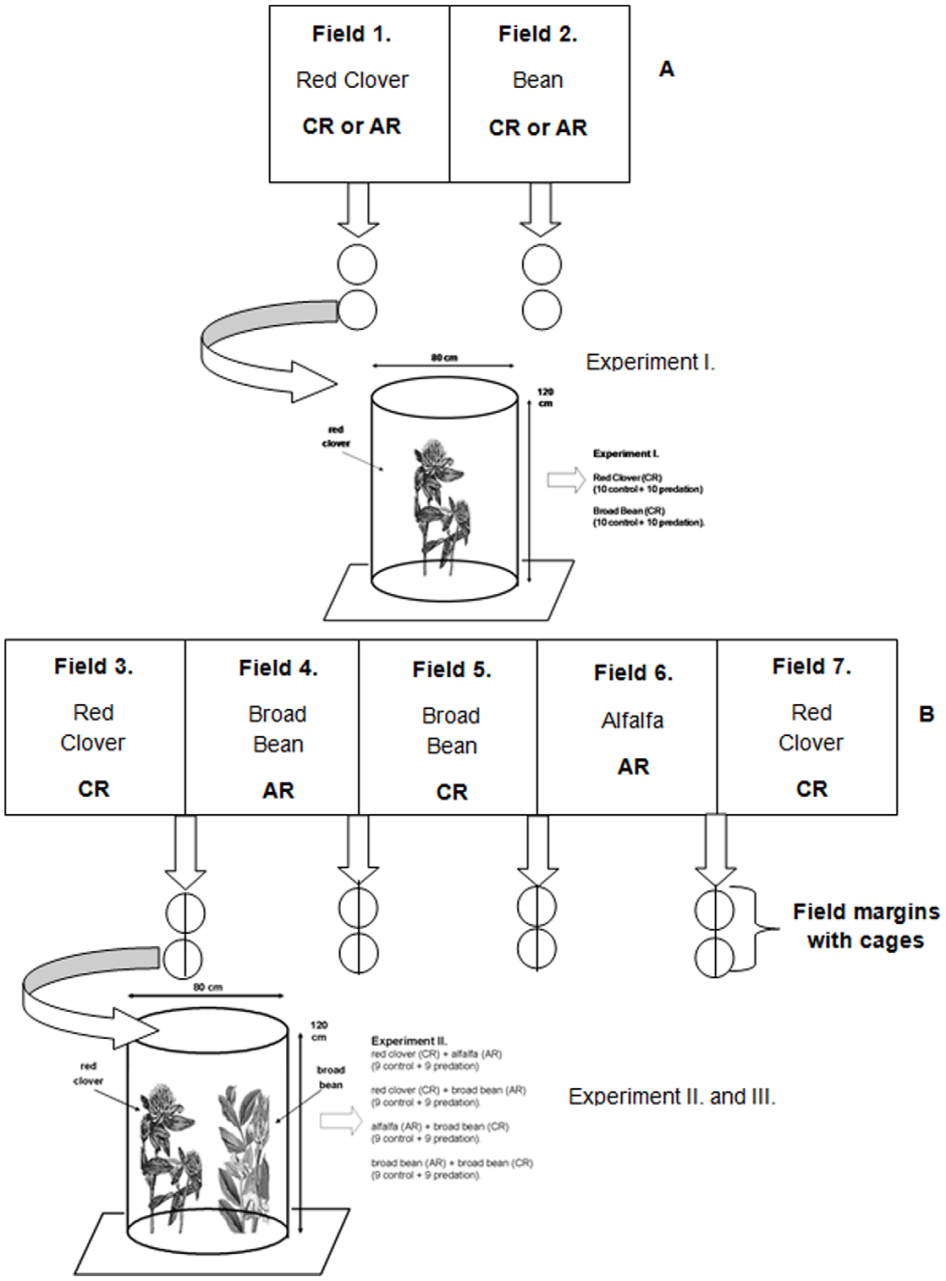
A simple landscape mosaic of red clover alfalfa and broad bean and the experiment design in Experiment I to test the relative fitness (per capita reproduction) and predation rata of CR and AR on broad bean, red clover and alfalfa (A) and a more complex and realistic landscape mosaic in Experiment II to test the potential fitness consequences of predation on pea aphid host races (B). Cages in Experiment I were placed in the middle of the fields and isolated single plant species. Cages in Experiment II were placed on the border of two adjacent fields thereby including two host plants. The cage design was similar in both experiments. CR – pea aphid clover race, AR – pea aphid alfalfa race.

We conducted three experiments to test whether predation is responsible for differences in fitness of pea aphid host races in an agricultural mosaic. In the first experiment we examined the relative performance (reproduction) of CR pea aphids on broad bean (*Vicia faba*) and red clover (*Trifolium pratense*) and then the performance of AR pea aphids on broad bean and alfalfa (*Medicago sativa*) in the presence and absence of predation (Figure 1A). However, this simple experimental context in which two host plants are spatially juxtaposed with each other doesn't reflect the agricultural environmental milieu. Clover is typically grown in an agricultural mosaic with alfalfa and broad bean (Figure 1B). We therefore conducted a second experiment to assess the fitness consequences of predation in a more complex agricultural field setting (Figure 1B). This second assessment was crucial given that predation effects on CR in natural agricultural settings could be mediated by the existence of a second pea aphid host race specializing on alfalfa (AR). Most studies only test for apparent competition in one direction [29], so the potential existence of asymmetry in apparent competition is not clearly evaluated for many species. Moreover, predator preference for CR or AR on different host plant contrasts may depend on the relative densities of pea aphid races on different host plants. Therefore we conducted a third experiment in which predation on CR and AR was evaluated under low and high densities.

## Materials and Methods

### Study system

Closely related perennial and annual leguminous plants (i.e. clover, alfalfa, bean and pea) are often planted in close proximity in mosaics of different sizes. This adjacency may have direct influence on predation rate of a widespread herbivorous species such as pea aphid. Lady beetles are generalist predators typically present in these agricultural mosaics and feeding in pea aphid colonies. Their predation level might differ between aphid host plants and thus create the conditions needed to cause differential aphid fitness among different host plants. Pea aphids have complex life cycles that include all-female, parthenogenetic generations that alternate with sexual generations. Each female gives birth to 50–100 living young at the rate of 6 or 12 a day. There are up to 20 or more generations a year. Pea aphids provide some of the best studied instances of host-plant adaptation and has repeatedly generated host-specialized populations adapted to both crops and wild legumes [4], [22], [29]. Specialization and differentiation have been most closely studied in populations from red clover and alfalfa [8], [22], [30].

### Ethics Statement

Aphids for rearing were collected as a part of a pest-management program that local farmers used to control aphid damage on agricultural fields. We were not responsible for, nor executed the pest-management program. Before aphid collection, farmers were contacted and permission obtained to enter their fields. For insect collection, no permits were required since the area where pea aphids and lady beetles were collected did not contain any strictly protected areas. Permits were also not required to use insects for experiments due to the observational nature of the data collection. All animal work was conducted according to relevant national and international guidelines. All aphids and lady beetles were released after the experiments.

### Field assessment, collection and rearing of aphids and predators

CR and AR pea aphids used in experiments were collected during the spring and summer from four different clover and four different alfalfa fields. Fields were located between 5 and 270 km from each other. Adult aphids were sampled during May four times each to have genetically mixed individuals from the same host species, thereby reducing the possibility of similar susceptibility to biotic and abiotic factors due to possible local adaptation. Prior to experimentation, aphids were reared in low-density populations to avoid stress by overcrowding on host plants (red clover, stock name Starfire Red Clover and alfalfa stock name Attention II) and on broad bean (stock name The Sutton). Host plants were grown individually in 15-cm diameter and 20 cm deep plastic pots under controlled greenhouse conditions with 16 h light, 8 h dark, 25°C, and 75% relative humidity to simulate the normal summertime climate. Altogether, 20 host plants from clover, 20 from alfalfa, and 40 from broad bean were used to rear aphids. Host plants were potted individually and pots were placed in dishes and watered by filling the dishes. This allowed water to be drawn upward through holes in the bottom of the pots, thereby preventing disturbance to aphids on plants. Ten host plants were isolated from each other in two blocks to avoid mixing races, one containing CR on clover and bean (20 clover and 20 broad bean plants) and the other AR on alfalfa and bean (20 alfalfa and 20 broad bean plants). Aphids were controlled for age by removing the adults after three days of reproduction. The offspring were reared until they had just molted into adulthood and then they were transferred to new plants where they could reproduce for another three days and were then taken off the plant. This procedure was repeated again and aphids from the next generation were used for experiment. This method (‘split-brood-design’ [31] allowed having aphids of similar age and possible lifetime for the experiment. To avoid the appearance of winged morphs, only good quality young plants were used and some individuals from each population were collected and released periodically to prevent overcrowding in host plants. The whole rearing process lasted approximately 30 days until there were enough un-winged similar-aged adult aphids to be used for the experiment. CR consisted about 50% red and 50% green morphs while AR 40%red and 60% green morphs during field collection. This color morph ratio was maintained throughout the experiment.

During and after aphid collection, assessments were carried out daily for three-week periods in clover, alfalfa, and two bean fields to identify the dominant predator. Six plots/field of ∼6 m^2^ each was randomly established. Inside each plot, another six subplots of ∼1 m^2^ each were established and sweep netted. All predators were counted by separately evaluating the most frequent species. Two-spotted lady beetles (*Adalia bipunctata*) were the most frequent predator during the whole assessment period, with numbers reaching a maximum of 50 individuals in one subplot. For experiments, lady beetles were obtained from the same fields (from two alfalfa, two clover, and two broad bean) 48 h prior to use in cages. To identify males and females, all individuals from the same field were kept together for 24 h. If mating between two individuals was observed, they were isolated together in a separate plastic dish and used together (male+female) in cages for the experiment. This avoided biased predator effects because predation level of males and females may differ; in particular, mated females are considered to have the higher appetites than males when forming eggs.

### Experiment I

This field experiment was performed using CR, AR and its lady beetle predators to test the relative performance of aphids in a simple agricultural mosaic. The experiment was conducted using red clover, alfalfa and broad bean host plants. Host plants were seeded at the end of April at normal crop density. The experiment started in the middle of June when host plants were approximately 40–45 cm high. Before the experiment, all other insects were removed from plants, after which plants were caged with low (0.2 cm) mesh-size net allocated at least 5 m from other blocks (Figure 1A). There were 10 plants in each cage. Cages were 80 cm wide and 120 cm high (Figure 1A). Over the next two days, any other insects observed in the cages were removed by hand. Thereafter, host plants inside cages received six wingless adult aphids assigned randomly into replicate blocks as follows (Figure 1A):

- Red clover with CR (10 control cages and 10 cages with predator).
- Broad bean with CR (10 control cages and 10 cages with predator).
- Alfalfa with AR (10 control cages and 10 cages with predator).
- Broad bean with AR (10 control cages and 10 cages with predator).

Each plant was examined after one day (when each adult had given birth to 8–10 offspring). If no reproduction or dead females were observed, then individuals were replaced. Aphids were allowed to reproduce for three days, having approximately 160–180 offspring in each cage. In ten treatment cages, two adult (female and male) lady beetles were introduced and the cages were closed again. Ten cages from each combination were kept free of predators and used as the control. One day later, all cages were carefully checked. Any plants that were attacked by ants from the ground (two cages from red clover predation, two from control and three from broad bean control) were excluded from any further assessment. Cages were examined every second day until the end of the 12-day experiment because at that time, female beetles laid eggs and new emerging lady beetle larvae would bias the aphid predation rate. Therefore, once eggs were observed (first time after 10 days) the cage was removed and the entire host-plant plot cut and brushed into a white plastic dish. The experiment was run for 10 days during which time none of the original offspring reached adulthood and started reproducing. In none of the cages the adult density overreaches the initial stoking number of adults at the end of the experiment; however we found relatively high number of fourth instar pea aphid nymphs. All initial predators survived until the end of the experiment. Aphid population density was counted by assessing separately adults and offspring on the entire host plant.

### Experiment II

The second field experiment was conducted using the host plants alfalfa, red clover, and broad bean in a typical row-crop agriculture planting (Figure 1B). The experiment evaluated the performance of CR on clover and bean. However, predation effects on CR in natural agricultural settings could be mediated by the existence of a second pea aphid host race specializing on alfalfa (AR) but also the broad bean host. We therefore evaluated the performance of CR and AR on their respective host plants in the absence and presence of their two-spotted lady beetle predator. Cages identical to those used in Experiment I were placed on the border between fields containing two host plant species at the same time in the following combinations (Figure 1B):

1. Red clover with CR +Alfalfa with AR (9 control cages and 9 cages with predator).
2. Red clover with CR +Broad bean with AR (9 control cages and 9 cages with predator).
3. Alfalfa with AR +Broad bean with CR (9 control cages and 9 cages with predator).
4. Broad bean with AR +Broad bean with CR (9 control cages and 9 cages with predator).

These host plant combinations were selected because this mosaic pattern of cultivated leguminous plants is the most frequent in both US and Europe. Host plants within cages were separated by approximately 20 cm to prevent aphids from moving between host plants. Pea aphids feed by penetrating plant organs with their stylets in search of vascular tissues [8] and movement between host plants or even between leaves of the same plants is low or nonexistent. A similar protocol to that of Experiment I was used: host-plant pairs (10 plants each) received six wingless adult aphids each and were assigned into replicate blocks as follows (Figure 1B).

Nine cages from each combination were assigned as treatment and two adult (females and males) lady beetles were placed on host plants inside each cage and left to move and feed. Nine cages from each combination were kept free of predators, and used as the control. Any plants that were attacked by ants from the soil were excluded from any further assessment. Cages were examined every second day until the end of the experiment. During this time the initial ratio of color morphs (50–50% red and green for CR and 40–60% red and green for AR) was assessed to evaluate potential movement between host plants. We observed the same color combination ratio throughout the experiment including the final number of offspring. All predators survived the experiment and during routine checks we observed predation on aphids. Because of cage exclusion (one from each host plant combination except broad bean+broad bean where we excluded two from control and one from predator treatment), we balanced the number of replicates for the predation treatment and control before the final assessment was made by randomly selecting six blocks of each host-plant combination. Again, once beetle eggs were observed, the cage was removed and the entire host plant plot was cut and brushed into a white plastic dish. This experiment also ran for 10 days during which time none of the original offspring reached adulthood and started reproducing. Aphid population density was counted by assessing separately adults and offspring in the entire host plant.

### Experiment III. Density dependent predation on CR and AR

We conducted the third experiment using the same protocol as experiment II, but with different starting densities of pea aphids. Host plants were assigned in replicates as described above. Different starting densities of CR and AR pea aphids were placed on host plants in the following combinations:

- Red clover with 12 CR adult aphid+Alfalfa with 6 AR adult aphids (6 control cages and 6 with predator).
- Red clover with 6 CR adult aphid+Alfalfa with 12 AR adult aphids (6 control cages and 6 with predator).
- Red clover with 12 CR adult aphid+Broad bean with 6 AR adult aphids (6 control cages and 6 with predator).
- Red clover with 6 CR adult aphid+Broad bean with 12 AR adult aphids (6 control cages and 6 with predator).
- Alfalfa with 12 AR adult aphid+Broad bean with 6 CR adult aphids (6 control cages and 6 with predator).
- Alfalfa with 6 AR adult aphid+Broad bean with 12 CR adult aphids (6 control cages and 6 with predator).
- Broad bean with 12 AR adult aphid+Broad bean with 6 CR adult aphids (6 control cages and 6 with predator).
- Broad bean with6 AR adult aphid+Broad bean with 12 CR adult aphids (6 control cages and 6 with predator).

All blocks were caged as described above. We let the aphids reproduce for three days having approximately 300–320 individuals/host plant in high and approximately 160 individuals/host plant in low density populations. Six cages from each combination were assigned as the predation treatment and two adult (females and males isolated together as described) *A. bipunctata* were placed in each cages. Six cages from each combination were kept as a predator free control. Cages were examined every second day until the end of the experiment. During this time the initial ratio of color morphs (50–50% red and green for CR and 40–60% red and green for AR) was assessed to account for movement between host plants. We observed the same color combination ratio throughout the experiment including the final number of offspring. All predators survived the experiment and during routine check aphid consumption was observed. No cages were excluded from the assessment. The same protocol was repeated throughout the experiment. The experiment lasted 9 days during which time none of the original offspring reached adulthood and started reproducing.

### Data analyses

For all experiments we used the final count of aphids for fitness analyses. The total counts of offspring at the end of the experiment were divided by the initially stocked adult density to estimate fitness (per capita reproduction). Data from experiment I did not meet the assumption of normality. Therefore the nonparametric Kruskal-Wallis test was used followed by a Mann-Whitney test to compare CR and AR fitness in the presence and absence of predators. Data from Experiment II met the assumption of normality therefore paired t-tests were used to evaluate whether fitness differences arose within host races. For the experiment II, we conducted four independent contrasts to evaluate CR fitness changes due to predation when CR on either clover or bean is juxtaposed with AR on either bean or alfalfa. The fitness loss for both experiments I and II was calculated as: Fd = P*100/C where Fd is the % fitness difference between predator (P) treatment and control (C). Relative fitness loss was calculated as 1-Fd.

Data from experiment III did not meet the assumption of normality; therefore the nonparametric Kruskal-Wallis test was used, followed by a Mann-Whitney test to compare the CR and AR fitness with different starting adult densities in the presence and absence of predators. This was done by using comparisons of host races within same densities in host plants. The first comparison evaluated the performance of CR on clover and AR on alfalfa (separately low densities in the presence and absence of predator and high starting adult densities in the presence and absence of predators). The second evaluated the performance of CR on clover and AR on broad bean in the presence and absence of predator. The third evaluated the performance of CR on broad bean and AR on alfalfa, while the fourth evaluated the performance of CR and AR both on broad bean in the presence and absence of predators. Confidence limits of P≤0.05 were considered significant for all experiments.

**Figure 2:**
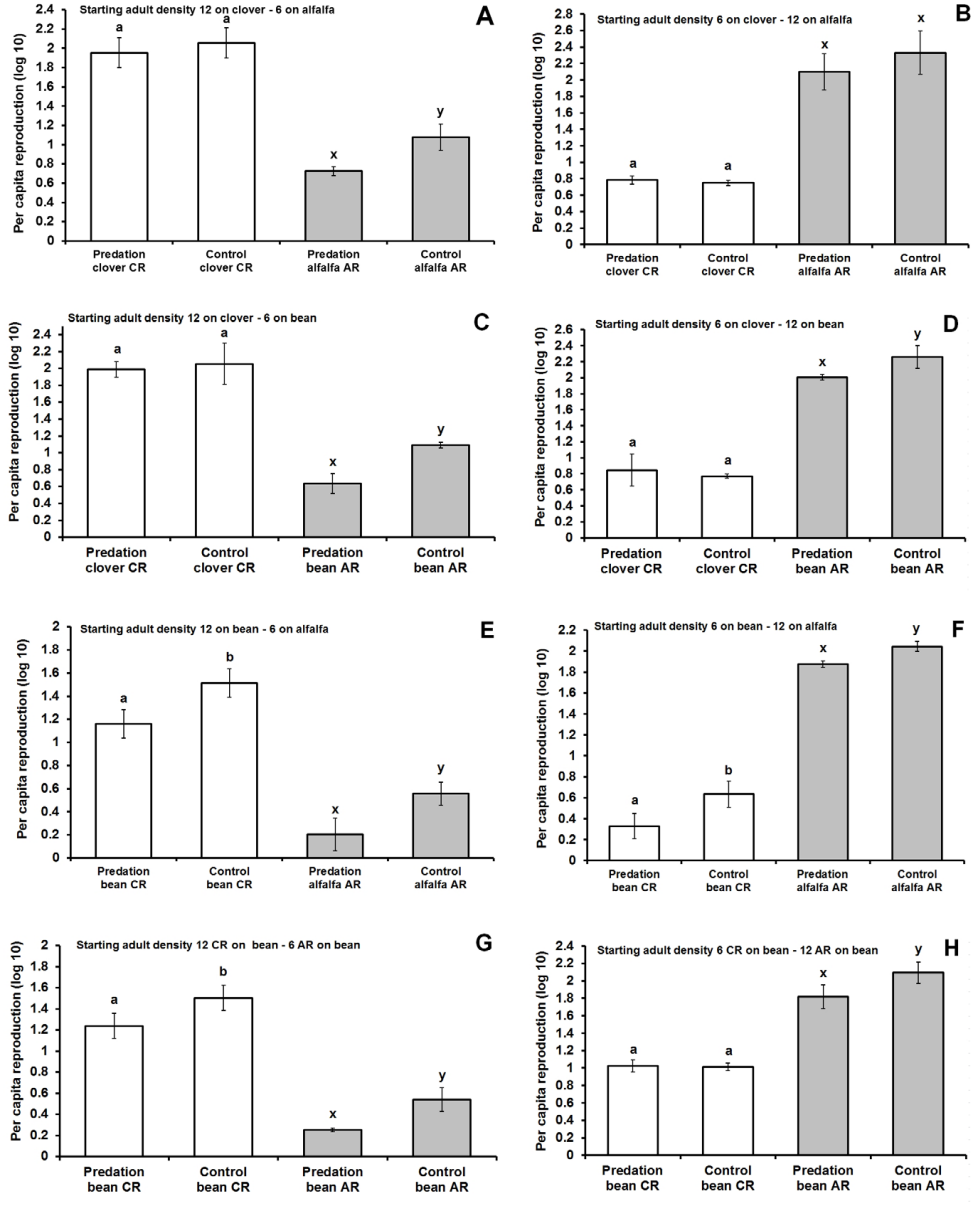
Effects of predation on CR and AR pea aphid fitness in different host plant when CR performing on clover in high density (12 starting pea aphid adult) versus AR on alfalfa in low density (6 starting adult pea aphid) (A), CR performing on clover in low density (6 starting pea aphid adult) versus AR on alfalfa in high density (12 starting adult pea aphid) (B), CR performing on clover in high density (12 starting pea aphid adult) versus AR on broad bean in low density (6 starting adult pea aphid) (C), CR performing on clover in low density (6 starting pea aphid adult) versus AR on broad bean in high density (12 starting adult pea aphid) (D), CR performing on broad bean in high density (12 starting pea aphid adult) versus AR on alfalfa in low density (6 starting adult pea aphid) (E), CR performing on broad bean in low density (6 starting pea aphid adult) versus AR on alfalfa in high density (12 starting adult pea aphid) (F), CR performing on broad bean in high density (12 starting pea aphid adult) versus AR on broad bean in low density (6 starting adult pea aphid) (G) and CR performing on broad bean in low density (6 starting pea aphid adult) versus AR on broad bean in high density (12 starting adult pea aphid) (H). White bars represent CR and grey bars represent AR. Different letters means statistical significant differences: “a” and “b” for CR and “x” and “y” for AR. Nonparametric Kruskal-Wallis test was used, followed by a Mann-Whitney test to compare the CR and AR fitness with different starting adult densities in the presence and absence of predators.

White bars on figures represent CR, grey bars represent AR. The per capita reproductions (fitness) in figures 2A–H were log10 transformed.

## Results

Experiment I revealed that within host plants CR fitness did not differ between predation treatment and control; however AR suffered significant fitness loss due to predation on both alfalfa and broad bean (Table 1). Experiment II revealed that predation caused no significant CR fitness reduction whenever CR was on clover and AR was either on alfalfa or bean. In these combinations, predation caused fitness losses of AR however (Table 1). Predation caused significant fitness losses whenever CR was on bean and AR was either on alfalfa or bean (Table 1). In these combinations, predation caused no fitness losses of AR however (Table 1).

**Table 1:** Effects of predation on CR and AR pea aphid fitness in different host plant contrasts within a simple (experiment I) and a more complex agricultural mosaic (experiment II).

	pea aphid host race			
host plant	CR fitness values	CR fitness loss	AR fitness values	AR fitness loss
	Experiment I (Mann-Whitney test)			
CR on clover	Predation: 122.4±6.5Control: 124.8±11.8	no fitness lossp = 0.96	-	**-**
CR on bean	Predation: 106±6.9Control: 103.2±4.1	no fitness lossp = 0.94	-	**-**
AR on alfalfa	-	-	Predation: 45±6.4Control: 110.55±13.7	**59.2% fitness loss** **p<0.01**
AR on bean	-	-	Predation: 78.44±1.5Control: 139.32±2.2	**43.6% fitness loss** **p<0.05**
host plant contrasts	Experiment II (paired t test)			
CR on clover vs. AR on alfalfa	Predation: 111±0.3Control: 113.3±0.2	no fitness lossp = 0.47	Predation: 93.99±0.4Control: 138±0.4	**31.8% fitness loss** **p<0.05**
CR on clover vs. AR on bean	Predation: 133.84±0.3Control: 137.6±0.3	no fitness lossp = 0.84	Predation: 57.6±3,4Control: 121.84±0.3	**52.7% fitness loss** **p<0.01**
CR on bean vs. AR on alfalfa	Predation: 43.66±4.4Control: 120.14±18.8	**63.6% fitness loss** **p<0.01**	Predation: 134.08±3.0Control: 135.6±3.7	no fitness lossp = 0.90
CR on bean vs. AR on bean	Predation: 57.88±3.1Control: 130.08±6.7	**55.5% fitness loss** **p<0.01**	Predation: 119.2±3.3Control: 137.84±3.8	no fitness lossp = 0.37

Fitness values represent the average number of offspring/female at the end of the experiment. The fitness loss was calculated by using the formula: 1- Fd = 1-(P*100/C) where Fd is the % fitness difference between predator (P) treatment and control (C). Data from experiment I were analyzed with Mann-Whitney test, data from experiment II were analyzed with paired t test.

Fitness loss of CR and AR varied with CR and AR starting density. There was no significant predator effect on fitness of CR on clover in either high CR vs. low AR density (CR p = 0.37) or low CR vs. high AR density (CR p = 0.99). However AR suffered significant fitness loss in high CR vs. low AR density combinations (AR p = 0.03) but no fitness loss in low CR vs. high AR density combinations (AR p = 0.24) (Fig. 2A, B).

CR did not suffer any fitness loss when on clover in high and low density combination with AR on broad bean (high CR vs. low AR density, CR p = 0.79; low CR vs. high AR density, CR p = 0.97), whereas AR suffered significant fitness loss in both cases (high CR vs. low AR density, AR p = 0.001; low CR vs. high AR density, AR p = 0.05) (Fig. 2C, D).

CR and AR in both high and low densities encountered significant fitness loss when CR was on broad bean in combination with AR on alfalfa (CR high density p = 0.001; AR low density p = 0.001; CR low density p = 0.001; AR high density p = 0.001) (Fig. 2E, F).

Fitness loss of CR and AR were significant when in high CR vs. low AR density on broad bean (CR p = 0.01; AR p = 0.001). Only AR suffered fitness loss when both races were on broad bean in low CR vs. high AR density (CR p = 0.72; AR p = 0.001) (Fig. 2G, H).

## Discussion

Insect host races are expected to arise when individuals are under different pressure, potentially leading to divergent selection via reproductive isolation [3], [7]. Typically, this is thought to arise because of a trade-off between the benefits of gaining food resources on a host plant and the cost of dealing with novel plant structures or chemicals encountered while searching for new hosts [24]. We show that different predator preference of CR and AR can also arise but fitness levels depend on environmental context, including associations of different host plants and aphid densities.

In combination, experiments 1 and 2 suggest that predation impacts fitness of CR aphids when CR aphids are on bean, but only when they are in the presence of AR aphids. Thus, for CR aphids, both host plant and the presence of competitors influence fitness (Table 1). This is generally consistent regardless of initial density of aphids or the host plant of competitors, except in the case where CR aphids are at low density relative to AR aphids and those AR aphids are also on bean. The environmental context under which predation influences AR fitness is different than that for CR. For AR aphids, predators have a substantial impact on fitness in all experimental conditions except where there were in the presence of CR aphids on bean and the starting density of CR and AR aphids was similar (Table 1, Fig. 2A–H).

Collectively, the experiments cannot provide definitive evidence for context-dependent predator-mediated apparent competition between CR and AR. This is because the possibility still exists that predation impacts on CR fitness on clover could be an increase in reproduction by the aphids, or it could simply be an interaction between the plant and aphid in the presence of the predator, with not real negative indirect interaction as a consequence of sharing a common natural enemy. According to our results there is clearly some interaction between host plant and aphid biotype in response to predation, however if this is an increased in reproductive potential of CR aphids when the predator is present this is apparent mutualism. In the case of CR aphids on bean however there is clearly a shift in predation to attacking CR aphids, which is apparent competition. Thus, the impacts of predation on aphid fitness are highly asymmetric and complex and dependent on both aphid genotype and environmental context.

Theory suggests that interactions in which the effect of one prey species on another is negative and the reciprocal effect is positive should be common [29], [32], [33], [34]. An example of this is the corn earworm (*Helicoverpa zea*), that clearly benefits from the presence of pea aphid but not vice versa: an asymmetric apparent mutualism [35]. Possible reasons for the asymmetry we observed between pea aphid host races may include: (i) different habitat complexity of host plant structure; and/or (ii) difference in trait-mediated effects [30]. The higher structural complexity (leafiness) of clover or alfalfa host plants relative to bean may influence natural enemy behavior through a trait-mediated indirect effect. Many natural enemies use chemical cues emanating from the host plant to search for prey and to locate suitable habitat patches [6], [36], [37]. In some cases the strength and the quality of the cue depends on the species of host plant utilized by the herbivore that could indicate a qualitatively higher prey [38]. This may be the case of a higher predator preference toward AR in alfalfa. AR may have higher food quality for predators. The strength and the quality of both CR and AR may be higher when host races exist on broad bean.

Moreover, broad bean excretes extrafloral nectar, which may attract predators. This may explain the high vulnerability of both races when on bean but not on their clover and alfalfa hosts (Table 1, Fig. 2A–H). The red and green color morphs may also exhibit different predispositions to engage in escape behavior (red morphs drop more frequently than green morphs when predators are present [39]. This may be because red morphs may be more sensitive to crowding than green morphs, with the consequence that there is a marked response of red morphs to ladybird kairomones associated with crowding [40]. In our experiment AR has 40% red and 60% green morphs, while CR had nearly 50% green and 50% red morphs. The asymmetry by susceptibility of colors in our experiment can be disregarded due to low divergences in color morphs between host races. Altogether we can conclude that for pea aphids, differential predator pressure can exist in a complex agricultural mosaic. The complexity of the agricultural mosaic can influence predators' choice of prey on different host plants that may have evolutionary consequences by increasing adaptation to host plants.
